# Familial Transmission of *emm12* Group A *Streptococcus*

**DOI:** 10.3201/eid2411.171743

**Published:** 2018-11

**Authors:** Rachel Mearkle, Sooria Balasegaram, Shiranee Sriskandan, Vicki Chalker, Theresa Lamagni

**Affiliations:** Public Health England, Chilton, UK (R. Mearkle);; Public Health England, London, UK (S. Balasegaram, V. Chalker, T. Lamagni);; Imperial College, London, UK (S. Sriskandan)

**Keywords:** Bacteria, Familial transmission, emm12, Group A Streptococcus, iGAS, Streptococci, Streptococcus pyogenes, Streptococcal infections, Post-exposure prophylaxis, Public health

**To the Editor:** We read with interest the recent research letter by Duployez et al. describing a cluster of invasive group A *Streptococcus* (iGAS) infections in a cohabiting couple in their 60s (*1*). The report illustrates the increased risk of infection for persons living in a household with someone with iGAS infection. We write to draw readers’ attention to our recent study, which adds to the body of evidence on the risk of household transmission of iGAS (*2*). 

Population-based studies from Australia, Canada, the United Kingdom, and the United States, based on 13 household clusters, assessed the risk of transmitting iGAS infection through household contact (*3*). We identified an additional 24 household clusters in England using addresses captured through national surveillance in 2009 and 2011–2013. For all 12 clusters in which *emm* typing was performed on both patients, results were the same for both. All secondary cases occurred within 1 month of the index case (median 2 days). Among contacts, the 30-day incidence rate was 4,520/100,000 person-years, 1,940 times higher than the background incidence (2.34/100,000 person-years). Spouses and partners ≥75 years of age (6 pairs) were at particularly high risk for developing infection; incidence was estimated at 15,000 (95% CI 5,510–32,650)/100,000 person-years, 1,650 times higher than the background risk in this age group (9.09/100,000, 95% CI 5,510–32,650). These data resulted in an estimated number needed to treat of 82 (46–417).

Duployez’s article also highlights differences between countries in policies for antimicrobial chemoprophylaxis. National guidance for public health management of community iGAS infection is being revised in the United Kingdom; oral penicillin V is currently recommended as the first choice for chemoprophylaxis (*4*). However, questions remain about the efficacy of chemoprophylaxis and the practicalities of timely administration to benefit others in a household, given that 38% of pairs were co-primary cases or had only 1 day between initial and subsequent infections. 
